# The Case for BPA as an Obesogen: Contributors to the Controversy

**DOI:** 10.3389/fendo.2019.00030

**Published:** 2019-02-06

**Authors:** Beverly S. Rubin, Cheryl M. Schaeberle, Ana M. Soto

**Affiliations:** Department of Immunology, Tufts University School of Medicine, Boston, MA, United States

**Keywords:** Bisphenol A, non-monotonic dose response, metabolome, adiposity, exposure windows, exposure route, perinatal

## Abstract

Since the inception of the term endocrine disruptor, the idea that the environment is an important determinant of phenotype has motivated researchers to explore the effect of low dose exposure to BPA during organogenesis. The syndrome observed was complex, affecting various endpoints such as reproduction and reproductive tissues, behavior, mammary gland development and carcinogenesis, glucose homeostasis, and obesity. This constellation of impacted endpoints suggests the possibility of complex interactions among the multiple effects of early BPA exposure. One key finding of our rodent studies was alterations of energy and amino-acid metabolism that were detected soon after birth and continued to be present at all time points examined through 6 months of age. The classical manifestations of obesity and associated elements of metabolic disease took a longer time to become apparent. Here we examine the validity of the often-mentioned lack of reproducibility of obesogenic effects of BPA, starting from the known environmental causes of variation, which are diverse and range from the theoretical like the individuation process and the non-monotonicity of the dose-response curve, to the very pragmatic like housing, feed, and time and route of exposure. We then explore environmental conditions that may hinder reproducibility and discuss the effect of confounding factors such as BPA-induced hyperactivity. In spite of all the potential sources of variation, we find that some obesogenic or metabolic effects of BPA are reproducibly observed when study conditions are analogous. We recommend that study authors describe details of their study conditions including the environment, husbandry, and feed. Finally, we show that when experimental conditions are strictly maintained, reproducibility, and stability of the obese phenotype is consistently observed.

## Introduction

The incidence of obesity and associated elements of metabolic disease has increased rapidly during the last 30–40 years (1–3) reaching epidemic proportions in the industrialized world. This phenomenon has stimulated scientists to ponder about novel environmental factors that could contribute to this sudden change (4). These include chemicals referred to as “obesogens” (5), that inappropriately foster lipid accumulation and adipogenesis and lead to obesity. Bisphenol A (BPA) is just one compound on a growing list of possible obesogens; however, due to the ubiquitous nature of BPA exposure, it may represent an important compound among the putative contributors to the obesity epidemic. In fact, BPA was detected in the urine of over 92% of a representative sample of the US population (6), and positive associations have been found between urinary BPA levels and several adverse outcomes, including obesity and associated elements of metabolic disease (7–9). In addition to a putative obesogen, based on the data available, BPA may also be considered a metabolic disruptor (10) and a diabesogenic compound (11).

There has been some level of controversy regarding the effects of BPA exposure on obesity and metabolic disease. The purpose of this review is to delineate potential factors underlying seemingly discrepant outcomes in animal studies. We posit that these factors encompass differences like the species and strain, developmental stage at the time of BPA exposure, the ages at which animals were examined, as well as the doses tested, the routes of exposure, and the environment surrounding the animals. Of particular importance is the shape of the dose-response curve, which for endocrinology is often non-monotonic regardless of whether the hormonally active agent is a natural hormone or an endocrine disruptor. In addition, there are theoretical reasons that can account for different responses.

### A Brief Theoretical Primer

In contrast to inert objects, living objects are not generic (interchangeable); they are individuals. Hence, what we measure as variation is the composite of an intrinsic and irreducible variation, since each animal is a unique individual, and a measurement error. Biologists address this problem by trying to make biological objects as “generic” as possible by using clonal cell lines and inbred strains, removing runts, culling litters, providing food of standardized composition, controlling the number of animals/cage, etc (12, 13). These procedures force a type of “invariance” or conservation into the system, allowing researchers to apply the mathematical tool of statistics. However, there is no mathematical theory of individuals, and thus biologists use statistics knowing that it has no way to contend with intrinsic variation. In addition, differences that are not statistically significant could be of utmost biological relevance. For example, Dolly the sheep was one clone that materialized from an enormous number of attempts. This also applies to the goat studied by Slijper that was born with paralysis of its front legs and soon learned to move by hopping on its hind legs. This behavioral accommodation resulted in dramatic morphological changes in bones of the hind legs and pelvis and changes in the morphology of the pelvic muscles (14) that were reminiscent of those present in bipeds. The mere fact that these singular results happen is sufficient to establish their relevance.

In this manuscript we analyze the effect of perinatal BPA exposure on adiposity and associated elements of metabolic disease and will show that when experimental conditions are kept constant to reduce variation (Table 1), the effect of BPA is reproducible. We also demonstrate that consideration of intrinsic individual variation allowed separation of two distinct phenotypes among females exposed perinatally to BPA (15).

**Table 1:** Our experimental practices for consistent conditions.

**Mice**	**Cages**	**#Animals/cage**	**Water**	**Diet**	**Exposure**	**Environment at the animal facility**
CD-1	Polysulfone	Culling at PND2: 4 ♀ and 4 ♂	Glass water bottles	Harlan Teklad, 2018; non-irradiated	Subcutaneous osmotic minipumps (Alzet)	Light cycle: 14–10
Charles River	Static racks to allow pheromone exchange	Weaning at PND21, separation of ♀ and ♂	Stainless steel sipper; rubber stopper	Each lot tested for estrogenicity levels prior to purchase	GD8 to PND16	Lights on at 4 a.m. EST
Supplier Facility: Raleigh until site closed, then Kingston	Corn cob bedding	4/cage at weaning; 2–4 depending on weight thereafter	Filtered water		0.025, 0.25, 2.5, 25, 250 μg BPA/kg BW/day	Temperature: 20–24°C
	Cotton enrichment		Water tested for estrogenic activity		Internal dose: <0.3 ng/ml BPA in serum	

## BPA as an Obesogen: Our Laboratory

We initially chose BPA as the model endocrine disruptor for study in our lab because of its estrogenic activity and its structural resemblance to diethylstilbestrol (DES). Therefore, we expected to find alterations of the reproductive system and reproductive physiology in offspring exposed to BPA *in utero* and during lactation, as had been described in the DES-induced syndrome (16). We found reproductive alterations, but the earliest difference we noted between BPA-exposed and control offspring was on body weight (17), an effect that had been reported 2 years earlier in female mice by Howdeshell et al. (18) and was later reported in mice exposed neonatally to DES (19).

### Body Weight and Adiposity

In the reproductive study mentioned above, we noticed differences in body weight in Sprague Dawley rats born to mothers that received BPA in their drinking water from gestational day 6 through weaning (17). Based on water consumption measurements, the exposure to the dams was estimated to be ~0.1 and 1.2 mg BPA/kg BW/day. The increase in body weight was modest, but significant. A similar study was performed by Somm et al. (20) using the same rat strain and only the lower BPA dose in drinking water. Both male and female BPA-exposed offspring were heavier at birth, and the females remained heavier through the termination of the study at 14 weeks. Increased perigonadal white adipose tissue weight and increased expression of adipogenic and lipogenic genes were observed in the females demonstrating that BPA exposure during gestation and lactation increased adipose storage and adipogenesis in a sex specific manner. Both BPA-exposed male and female offspring had increased body weights relative to controls when fed a high fat diet (HFD).

Subsequent studies in our lab using outbred CD-1 mice also examined the effects of perinatal BPA exposure on female reproduction and reproductive tissues (21–25). As in our previous rat study, we could not help but notice the increase in body weight in our BPA exposed mice. Also, when performing ovariectomies, we noted increased adiposity and ovarian fat pad size in the females born to mothers exposed to low levels of BPA. The BPA exposure for these studies was provided via osmotic minipumps that were implanted subcutaneously into pregnant females on GD-8 and released BPA through postnatal day 16; they provided continuous delivery of low levels of BPA (ranging from 0.025 μg/kg to 250 μg/kg BW/day) with great precision. Levels of unconjugated BPA in blood samples were below the detectability of the BPA assay (0.3 ng/ml) at all doses tested (15), and thus they are below or within human levels of exposure as measured in serum or plasma by analytical chemistry [adults: a range of 0–1 ng/ml [reviewed in Vandenberg et al. 26]; umbilical cord blood: median = 1.03 ng/ml (27).

In later studies undertaken to examine the effects of perinatal BPA exposure on body weight and adiposity, we continued to observe increased body weight and fat pad weights, adipocyte hypertrophy, and an increased number of white adipocytes in the intrascapular brown fat depot. Most recently we reported increased body weight and fat mass measured by echoMRI in male and female mice exposed perinatally to BPA. This outcome was exacerbated in females exposed to BPA both perinatally and peripubertally, particularly when exposures were 2.5 and 25 μg BPA/kg BW/day (15). The additional peripubertal exposure increased insulin resistance, fat mass, BW, and inflammation in females in a dose dependent manner. Although the males showed significant increases in body weight and fat mass with perinatal BPA exposure, they did not show increased detrimental effects with the additional peripubertal exposure. This suggests that the specific extended period of BPA treatment was more damaging in females, or that females were more sensitive to harmful metabolic effects of BPA during the peripubertal window of exposure. Evidence of inflammation of fat pads was observed in BPA exposed male and female mice. Also observed was lipid accumulation in liver (15, 28) and increased serum leptin levels (15).

### Metabolome Data

Metabolomics is the measurement of the set of small molecules (metabolites) that result from metabolism (29). The integration of metabolomics and physiopathological studies provides valuable information for understanding endocrine disruption and is expected to produce useful data for risk assessment of endocrine disruptors such as BPA. Metabolic fingerprints based on nuclear magnetic resonance (NMR) spectroscopy, can detect slight changes in the metabolome of cells, tissues, or organisms exposed to endocrine disruptors (EDs) opening the way to examine whether exposure to an ED results in global alterations of metabolism, whether these changes persist after cessation of exposure, and whether further changes arise in aging. Additionally, and in contrast with other omics, the interpretation of metabolomics is straightforward as most metabolic pathways are known, and metabolic network analysis can be used to understand how a given set of metabolites in a metabolomic profile are linked (30).

Metabolomics was used in our studies to examine global metabolites in our BPA exposed mice and rats (31, 32). In all cases, at all ages studied, metabolomic profiles differed in BPA exposed and control animals. Our first metabolome study involved male mice exposed to 0.025, 0.25, or 25 μg BPA/kg body weight/day from GD8 to PND16 (32). Aqueous extracts of PND2 animals and of livers, brains, and serum samples from PND21 animals were examined by 1H nuclear magnetic resonance spectroscopy. Endogenous metabolic fingerprints revealed changes in the global metabolism in PND2 extracts. Consistent with the findings of Alonso-Magdalena et al. (33) demonstrating that BPA exposure during fetal development in mice leads to disruption of glucose homeostasis that manifested at 6-months of age, the metabolomic profile in our study revealed that glucose was already affected by perinatal exposure to BPA at PND2. Additional alterations at PND2 included variations in pyruvate, amino acids, and neurotransmitters. Regarding PND21, statistical analysis successfully discriminated among treatment groups in liver, serum, and brain samples. Variations in glucose, pyruvate, amino acids, and neurotransmitters (γ-aminobutyric acid and glutamate) were identified. The fact that these alterations occurred well before the manifestation of the classical pathophysiological signs of metabolic disease suggests that metabolic pathways are an early target of BPA, rather than a consequence of obesity. Studies in female rats, also revealed alterations of energy, and amino-acid metabolism in the BPA exposed animals at all ages examined (31).

## Factors That Can Influence Effects of BPA Exposure on Body Weight and Adiposity

Although our studies in CD-1 mice have consistently suggested that BPA is an obesogen, there is considerable controversy in the literature that has caused many to question whether there is adequate evidence of the obesogenic properties of BPA. Several animal studies have noted increased BW and/or adiposity in their early BPA exposure models (15, 17, 18, 20, 34–43) while other studies have reported a decrease in BW and/or adiposity (40, 44–47) or no change (48, 49). Reviews of the data from human studies have also generated some controversy regarding the association between BPA exposure and obesity and metabolic disease (50–52). It is clear from the mouse studies in our lab that the effects of perinatal exposure to BPA on body weight, and fat mass, and associated parameters of metabolic disease are sex-dependent, dose dependent, and influenced by the precise window of exposure as well as the time of examination.

Route of exposure, diet, and housing conditions as well as other environmental factors undoubtedly play a role in the manifestation of increased adiposity and could potentially mask effects of BPA exposure. For example, accidental BPA exposure from damaged polycarbonate cages (53) led to meiotic disturbances including aneuploidy in oocytes in the mouse colony maintained by Pat Hunt. This finding demonstrates how external conditions could interfere with the ability to detect the effects of early BPA exposure on BW and adiposity or any other measurements. In order to facilitate comparisons of data from different laboratories, the factors mentioned above should be included in materials and methods (Table 2). Our practices for keeping the living conditions of our animals as constant as possible are summarized in Table 1.

**Table 2:** Conditions that should be made explicit in Materials and Methods sections of research papers.

Strain and supplier	Specify the supplier's facility where animals were raised wherever possible
Feed and supplier	Specify which type of diet (casein-based, soy-based, other?) catalog number and supplier
	Specify if feed was tested for estrogenicity^**^ and by which method
	Was the chemical of interest tested for its presence in feed?
	If the nutrient composition cannot be easily accessed, provide a short description (Calories from fat, % fat,% protein)
Water and water bottle	Filtered or tap?
	Glass or plastic bottle?
	If plastic what type of plastic?
	Stopper, sipper materials
	Was water tested for estrogenicity^**^?
Caging materials	What type of plastic? Was it tested for estrogenicity^**^?
	Static or ventilated racks?
	Enrichment? What materials?
	Bedding type (wood shavings, corn cob, etc)
	Number of animals/cage
Overall facility	Temperature range
	Humidity range
	Light/dark cycle
Animals	How many per litter
	Sex ratio
	Culled? To what number on what day
	Weaning day For Mice: exposure to pheromones by presence of males and females in alternate cages
Exposure	Route
	Period
	Dose
	Vehicle
	Internal circulating dose if possible
Timepoints	When were samples collected or measurements taken?

** *Estrogenicity testing is a good measure even when the ED tested is not estrogenic, because of the ubiquity of contaminants with estrogenic activity that could be preset in food, plastics and water. Estrogenic activity could affect metabolism and interfere with the development of obesity*.

### Gender

There are several reports of increased adipogenesis in BPA exposed females relative to males particularly at young ages (20, 43). We initially noticed increased body weight and fat mass in our CD-1 mice with females showing more of an increase than males; however, that finding was influenced by changes in the environment and in housing conditions. For example, when males were singly housed to facilitate measurements of food intake and metabolism, the male data showed less variability allowing a clearer pattern to emerge. Whether the decrease in variability was influenced by the absence of a dominance hierarchy typically observed in group housed males, remains to be determined. In contrast to the males, when females were individually housed at 8 weeks of age, many of the BPA exposed females showed a tendency to lose weight. Overall compilation of recent data revealed that increased body weight and fat mass were more consistently apparent in perinatally exposed male than female CD-1 mice. This finding may be attributed, in part, to the hyperactivity observed in a subset of our BPA exposed females that represents a strong confounding factor when studying body weight and body composition and will be discussed below (15).

A recent study by Desai et al. (42), reported significant increases in BW and fat mass at 3 and 24 weeks of age in male but not female Sprague Dawley rats born to mothers exposed to BPA in their drinking water from 2 weeks prior to mating through pregnancy and lactation (5 mg BPA/liter which provided 500–900 μg/kg BW/day during pregnancy and ~1500 μg/kg BW/day during lactation). Further study of the BPA exposed males at 3 weeks of age revealed an increase in adipocyte size, lipogenic factors (SrebP1 and C/EBP alpha), and inflammation in the retroperitoneal fat pad. Sex also has a large impact on the effects of early BPA exposure on elements of metabolic disease. This is particularly apparent regarding the regulation of glucose/insulin homeostasis which has been extensively documented by Nadal and colleagues (54–56). They have clearly shown effects of gestational exposure to BPA on glucose regulation in male offspring that is not apparent in their female siblings at the times examined (33). Wistar rat offspring born to mothers exposed to 50 μg BPA/kg BW/day from GD0–PND 21 (by oral gavage) showed increased body weight on a chow diet. When fed a high fat diet (HFD), the body weight of males was increased before females (9 weeks vs. 15 weeks). This pattern was also seen in serum insulin levels that were increased at 15 weeks in males and 26 weeks in females (34).

### Hyperactivity

As mentioned above, increased activity has been reported in rodents exposed perinatally to BPA (15, 40, 44). Hyperactivity represents a serious confound to the assessment of increased body weight and adiposity and metabolic parameters in BPA exposed offspring. Although others have reported hyperactivity in both BPA- exposed males and females (57–60), in our studies, hyperactivity was more extreme and far more prevalent in BPA exposed females. A subset of females showed flipping or running behavior; both are repetitive behaviors that served no obvious function but were persistent in the affected animals (15). They remained lean despite a significant increase in food intake. If their data were included with the other females, no differences were noted in mean BW and mean estimates of adiposity as there was too much variability to allow statistically significant findings. If the data from the flippers were eliminated as outliers, the remaining data showed a pattern of increased body weight and adiposity in the non-affected females (15). It will be important to identify the factors involved in causing hyperactivity in a subset of the BPA exposed female offspring and to determine how widespread this phenomenon may be in other laboratories. Of interest, in their studies of C57Bl6 mice, van Esterik et al. reported increased body weights in their BPA exposed males and not their females although they did mention increased activity in their females (40). Similarly, Anderson et al. (44), reported decreased body weight and adiposity in their BPA exposed A^vy^ mice that was most pronounced in females, and when their movements were tracked, the females showed clear evidence of hyperactivity (44). In a study by Ryan et al. (49), when examined at 9–14 weeks of age, perinatally BPA exposed female CD-1 mice (but not their male siblings) consumed more calories and had less body fat than control females. These data would be consistent with increased activity levels or increased energy expenditure in the BPA exposed females. Hyperactivity was not measured in this study, and it can easily be overlooked as the hours of observation typically fall during the light cycle when the animals are sleeping.

Early BPA exposure has been found to alter cortical organization (61) and change dopaminergic projections in the neocortex which may play a role in activity changes. In our studies it is not clear why a subset of the females with identical BPA treatments show pronounced hyperactivity while others including their female siblings do not. This is an important observation to pursue and understand, particularly when one is attempting to study obesity and associated metabolic disease as it will have profound effects on the interpretation of the compiled data. Consistent with the results in rodents discussed above, it should be noted that there is a growing body of evidence suggesting an association between urinary BPA levels and increased activity in children (57, 62, 63).

### Diet

We have routinely used a soy protein-based diet for our studies (Harlan-Teklad 2018). The estrogenicity of all food batches are tested in our laboratory prior to purchase using the ESCREEN assay (64). Only those found to have minimal estrogenic activity (<20 pmol of estrogen equivalents per gram) were purchased. Based on evaluation of available studies in the literature, casein based diets appear to be less likely to show the effects of BPA on several parameters (65) including body weight and adiposity (44, 48, 49, 66). Increased body weight and fat mass in BPA exposed rodents tended to be observed in animals eating a soy-based diet and not necessarily in a casein diet (44, 48, 49, 66). Ruhlen et al. have reported that a casein based diet elevated body weights of control animals and could therefore mask the effects of BPA on BW and adiposity in mice (65). In contrast, Cao et al. have reported that in their study in rats, it was soy and not BPA that caused increased body weight (48). However, in our studies with soy-based chow, we saw consistent effects of BPA. Unfortunately, many studies in the literature fail to provide information on diet; these omissions make it difficult to compare data between studies to formulate accurate conclusions.

### Exposure Level: Low Doses, Monotonic and Non-monotonic Dose Response Curves (NMDRCs)

Exposure level is very important when studying the effects of early BPA exposure. BPA does not show a monotonic dose response curve for many endpoints including effects on BW and adiposity. The importance of the BPA dose on body weight and adiposity is clearly illustrated in a study by Wei et al. in rats (34). In that study, the lowest BPA dose administered by oral gavage through gestation and lactation (50 μg/kg BW/day, the current EPA reference dose) was obesogenic and predisposed the animals to elements of metabolic disease. The two higher doses (250 and 1250 μg/kg BW/day) did not produce these effects. Therefore, if only the higher doses had been examined, the report would conclude that BPA had no effect on BW, adiposity, and associated elements of metabolic syndrome. In another study in male CD-1 mice, animals were provided with 1 of 5 doses of BPA during the period of preadipocyte differentiation (GD9-GD 18) ranging from 5 μg to 5,000 μg/kg BW/day (35). The lower doses were more likely to increase BW and adiposity including adipocyte number and adipocyte size. In fact, no significant effects on these parameters were noted at the highest dose. Of interest, the studies of Tyl in mice (46) and in rats (47) revealed a very significant decrease in body weights in males and females exposed to the highest levels of BPA (500 mg or 600 mg/kg BW/day) during development. These levels were extremely high relative to the current EPA reference dose (67) and would not be considered environmentally relevant exposure levels. The toxicological model that presumes that the highest doses will provide the greatest effects is not typically applicable to endocrine disruptors or natural hormones.

A dose-response curve plots the intensity of a given effect over a range of doses examined. A monotonic response is characterized by a slope that does not change sign. In contrast, a non-monotonic dose response curve is characterized by a slope that changes sign within the dose-range tested. Some curves are U-shaped, others have an inverted U-shape and in others, the sign of the curve may change in multiple points along the range of doses examined. Non-monotonic dose-response curves are frequently seen in endocrinology. They range from proliferative effects observed in cell culture studies to whole organ, organismal effects and are even observed in epidemiological studies (68, 69).

Some processes leading to non-monotonicity have been identified in cell culture. Natural hormones like estrogens (70) and androgens (71–73) affect proliferation in a biphasic dose-response. At low doses, for example, sex steroids induce cell proliferation by neutralizing a plasma-borne inhibitor. At high doses, sex steroids block cell proliferation by inducing intracellular inhibitors (74) or by inducing cell death (75).

Natural hormones and endocrine disruptors also display non-monotonic dose-response curves when assessed *in vivo*. The vom Saal group observed non monotonic effects of estradiol and DES on prostate weight (76). Our group demonstrated that estradiol produces a monophasic dose response curve when the endpoint measured is gene induction or the expression of a protein. In contrast, at higher levels of organization the uterus displays a monotonic dose–response whereas the mammary gland exhibits a non-monotonic response to increasing levels of estradiol (77). From a theoretical point of view, monotonic phenomena can be easily modeled due to their simplicity, by assuming that each step in a linear pathway behaves according to the law of mass action (78); thus, high doses will give predictable high effects. On the contrary, modeling a non-monotonic effect is not possible without first obtaining results from experiments using a complex experimental design to separate the components of such NMDRCs.

### Exposure Window

Exposure windows must be considered when reviewing data on early exposure to BPA. Liu et al. published a study in C57Bl6 mice (38) in which the BPA exposure windows were dissected to include the following time intervals: GD 1-6; GD6-PND0; PND0-PND21; and GD6-PND21. Gestational exposure from GD6 to birth resulted in decreased body weights in males and females, and the postnatal exposure from birth to weaning resulted in a significant increase in body weight in the males. In contrast, for glucose tolerance and for insulin tolerance, the most consistent and persistent problem was seen in animals exposed from GD6 to PND0. This study delineates distinct differences in the exposure windows that affect BW and glucose homeostasis. These windows are reminiscent of the work of Cederroth and Nef (79) who observed that the beneficial effects of dietary soy and phytoestrogens on adiposity were noted only in postnatally treated CD-1 mice whereas improvements in glucose tolerance were restricted to animals that had fetal exposure.

When considering obesity and metabolic disease, it is important to realize that the metabolic circuits for food intake and metabolism in the hypothalamus are established primarily after birth. Therefore, neonatal BPA exposure could influence the development of these important circuits. The exquisitely timed postnatal leptin surge is essential for the proper development of neural circuits for food intake and metabolism (80), and MacKay et al. have provided evidence that peak leptin secretion is delayed in mice exposed perinatally to BPA (81). Disruption to the timing of the leptin surge impaired development of the melanocortin system resulting in alterations in the hypothalamic feeding circuitry that could contribute to metabolic disturbances in adulthood.

### Route of Exposure

The route of exposure to BPA may have a significant effect on the outcomes observed. Although diet is considered the main source of BPA exposure in humans and ingestion the main route of intake, it is not the only source of exposure (82, 83). BPA exposure can occur through food and water, dermal absorption as occurs with BPA-laden register receipts (84–87), and via inhalation of air and dust. Moreover, BPA levels remain measurable in fasting individuals (88) suggesting the potential for non-oral sources of exposure, longer half- life, or storage in the body that differs from the idea of a relatively rapid clearance rate for this compound following ingestion. Of interest, urinary BPA excretion was elevated 84% in volunteers who handled thermal receipts and the delayed return to normal pre-exposure levels suggested a potential difference in bioavailability of BPA following dermal absorption relative to oral exposure (89). Some suggest that the non-oral exposures, although they are considered to occur at significantly lower levels, may be more potent as they avoid the first pass metabolism of BPA by conjugation and therefore dermal sources may have far higher toxicological relevance than expected (83). In a controlled experiment, handling of cash register receipt paper plus still wet hand sanitizer elevated unconjugated BPA serum levels (84) to those found after oral exposure to 50 μg/kg BW (90). Dermal contact may be the main contributor when both unconjugated and conjugated BPA are considered (82).

Exposure routes for BPA differ widely in the numerous studies reported. Whether animals receive their BPA once a day as a bolus or throughout the day could conceivably alter the impact of the same daily dose. Studies that use a single daily dose have provided BPA in a single subcutaneous injection, or by oral gavage, or less intrusively, in a drop of oil on the tongue or dispensed on a cookie. Although most studies aim to provide BPA levels in the range of human exposures, humans are not exposed to a single daily dose of BPA. In contrast, other studies provide BPA throughout the day by incorporating it into the food or the drinking water or via silastic capsules or osmotic minipumps. Although they may afford the same daily dose as that provided by bolus delivery, these more continuous or intermittent exposure paradigms could have a different effect on the animal (91, 92). In most of our studies, animals were exposed to continuous low levels of BPA via osmotic minipumps. This method allowed us to provide very low BPA doses (beginning at 0.025 μg BPA/kg BW/day) with a precision that would not be technically possible in other non-bolus dosing regimens.

In one study that compared subcutaneous and oral exposure of BPA, neonatal male rats (3-day old) received 10 μg BPA/kg BW in oil. The same preparation was administered as a subcutaneous injection or as an oral bolus. As expected, the maximal serum concentration of free and total BPA was higher with subcutaneous administration (91). A second study compared an oral bolus of BPA with administration via diet in mice. The authors reported different patterns of BPA concentration. Bolus administration was found to peak faster (1 h) than administration through food (6 h); however, bioavailability in the form of unconjugated BPA was higher when administered in food (92). Therefore, the route of administration may have significant effects on study outcomes.

### Age of Outcome Measurement

Many effects of early BPA exposure do not manifest until later in life while others are apparent early in life. For example in the study by Somm (20), both male and female pups exposed to BPA *in utero* were heavier at birth than controls; however, at PND 21, only the females remained significantly heavier. Both males and females were heavier than controls when fed a high fat diet. Ryan et al. (49) found that males and females were heavier at weaning, but the increase in BW did not persist through week 14 when the study was terminated Several studies end at weaning or soon after and report no differences in BW or adiposity (45), but it is possible that such findings might have been observed in adulthood if they were examined. Malaise et al. reported (93) that relative to controls, mice exposed perinatally to BPA (50 μg/kg BW/day) were significantly leaner at PND 25, and they had decreased gonadal fat at PND 45. However, additional measurements from PND 70 to the end of the study at PND 170 revealed a significant increase in body weight and adiposity in BPA exposed mice relative to controls. Similarly in the study by Wei et al. (34), rats exposed perinatally to BPA (50 μg/kg BW/day) showed significant increases in body weight on a standard chow diet beginning at 17–19 weeks of age.

## In Summary

The purpose of this article was to examine the controversy surrounding BPA as an obesogen. To address this issue, we focused on obesity and adiposity and only peripherally touched on its metabolic effects on glucose homeostasis and lipid metabolism, as these subjects are addressed by other articles in this issue. To assess the controversy, we focused on perinatal exposures in rodents since our own data from this window of exposure could be used to illustrate consistent results over 10 years of attempting to maintain a constant environment. Moreover, we found that departing from these stringent conditions resulted in measurable changes.

If one examines the existing publications on exposure to a relatively “low dose” of BPA during a window that includes gestational and lactational exposure in rodents, most references show some indication of increased BW and/or increased adiposity and/or elements of obesity-associated metabolic disease. This is true even though the endpoints collected, and the windows of observation may be diverse. Some papers not showing such an outcome reported potential confounds that could preclude increased body weight and adiposity such as hyperactivity, a feature that may go undetected unless animals are observed close to the start of the dark cycle. We also found that the trajectory of increased body weight and adiposity was more clearly defined when males were singly housed rather than group housed. Diet appears to be another contributor to phenotype. A review of the literature suggests that animals exposed to a soy-based diet were more likely to reveal the obesogenic properties of BPA than those fed a casein-based diet. Moreover, in some studies, obesogenic properties of BPA were enhanced or brought out by feeding a HFD. From our overall analysis, we posit that these interactions may explain the lack of concordance among apparently similar experiments, pointing to the need for investigators to publish detailed descriptions of housing, diet, caging materials, culling practices, and other crucial methodological details that are often omitted from the materials and methods (Table 2).

One striking result from our metabolomic studies is that as early as 2 days after birth there are clear changes in amino-acid and energy metabolism, including glucose in the BPA exposed animals. These alterations precede the clinical manifestations of obesity and associated elements of metabolic disease including altered glucose homeostasis. Differences in the metabolic fingerprints between control and BPA exposed offspring persist through life. The precocity of altered metabolomic profiles suggest that they may play a causal role in the manifestation of elements of metabolic disease in adulthood.

The shape of the dose response curve may sometimes explain divergence between two studies, because non-monotonicity is a frequent feature of BPA effects. If the effect studied has a non-monotonic dose response curve, and only one or two BPA doses are tested, it is likely that one or more of the multiple effects of BPA could be missed. It is expected that because BPA targets distinct receptors and various organs the resulting syndrome would be complex (94). However, it is important to keep in mind that animals are individuals, and therefore, not all animals will be affected identically. If one considers all the sources of variation described above, the fact that obesity and/or alterations of metabolism are observed in the majority of studies with some equivalency of exposure times and levels argues for the robustness of the effect.

Although we reviewed studies of early (fetal and neonatal) BPA exposure here, studies of BPA exposure in adult rodents have revealed effects on adiposity and elements of metabolic disease (95–97). One particularly interesting finding was observed in females exposed to BPA (10 or 100 μg/kg BW/day) during days 9–16 of pregnancy (33, 95). When examined on days 16–18 of pregnancy, females exposed to BPA revealed glucose intolerance and elevated levels of plasma insulin, triglycerides, and leptin relative to controls (33). By 4 months postpartum, BPA exposed mothers had increased body weights and at 6.5 months they had increased body weights, and increased perigonadal fat pad weights (95). Alterations in glucose and insulin tolerance were observed by 4 months postpartum, worsened at 5 months, and became even more pronounced at 6 months. In contrast, non-pregnant females exposed to the same level and length of exposure to BPA showed no changes in glucose or insulin tolerance when examined at 4, 5, or 6 months after treatment. These data reveal harmful long- term implications for BPA exposure during pregnancy.

Epidemiological studies have found a positive association between urinary BPA levels and obesity and diabetes in adults, (7–9, 98–100) children, and adolescents (101–108); others have questioned the strength of the association (50, 51). Of particular interest, a recent and rare planned exposure study in humans provided oral administration of the reference BPA dose to volunteers and measured the insulin/C-peptide response to administered glucose. Although this study had a small sample size, BPA exposure was found to alter this response (90).

What are the important messages that one can glean from these studies? The most urgent one concerns public health. The studies addressed here bolster the notion that BPA is an obesogen and metabolic disruptor. The incidence of obesity and obesity-associated metabolic disease including diabetes is increasing to alarming proportions, and human data shows a positive association between BPA level and health effects (109). Given the fact that BPA exposure is ubiquitous, we need to conclude that BPA is a public health concern and take action to limit our exposure. European countries are reducing exposure by banning BPA in food packing materials, and a new law in France will ban the use of plastic in direct contact with food in school cafeterias (110).

A second issue in need of exploration is how BPA triggers these effects. The biological explanation of the syndrome produced by early BPA exposure is undoubtedly complex. It is worth remembering that the path from receptor binding to phenotype is not an obvious straight line. This complexity suggests it will take a long time to determine precisely how the BPA phenotype is determined. Organogenesis involves more than molecular interactions. As organs form, we observe supracellular phenomena such as cell to cell interactions, electrical gradients, mechanical forces, cell migration, and more (111). Additionally, the external environment co-constructs the phenotype (112) and the factors discussed above can modulate and affect the response to BPA. Therefore, we need to explore multiple pathways and processes that include all relevant levels of biological organization and we need to carefully control and report the conditions of our animals in the process. Unraveling the complexity of the BPA syndrome will provide us with a global understanding of how a very simple molecule derails development creating phenotypes prone to the development of multiple pathologies.

## Author Contributions

All authors listed have made a substantial, direct and intellectual contribution to the work, and approved it for publication.

### Conflict of Interest Statement

The authors declare that the research was conducted in the absence of any commercial or financial relationships that could be construed as a potential conflict of interest.
